# Maskless Synthesis of van der Waals Heterostructure Arrays Engineered for Light Harvesting on Large Area Templates

**DOI:** 10.1002/smll.202400943

**Published:** 2025-03-05

**Authors:** Matteo Gardella, Giorgio Zambito, Giulio Ferrando, Lorenzo Ferrari Barusso, Rajesh Chennuboina, Luca Repetto, Matteo Barelli, Maria Caterina Giordano, Francesco Buatier de Mongeot

**Affiliations:** ^1^ Dipartimento di Fisica Università di Genova Via Dodecaneso 33 Genova 16146 Italy; ^2^ Present address: CNR‐IMM Unit of Agrate Brianza Via C. Olivetti 2 Agrate Brianza MB 20864 Italy

**Keywords:** 2D‐TMD semiconductor layers, flat optics, maskless nanolithography, MoS_2_, photon harvesting, van der Waals heterostructures, WS_2_

## Abstract

Large area stacking of van der Waals heterostructure arrays, based on 2D Transition Metal Dichalcogenide semiconductors (TMDs), is achieved by an original physical deposition process utilizing Ion Beam Sputtering. Silica substrates endowed with periodically faceted nanoridges are fabricated using interference lithography and serve as templates for maskless deposition of TMD at glancing angles. This approach enables the creation of laterally confined few‐layer WS_2_ nanostripe arrays coated by MoS_2_ films. The subwavelength periodicity of the high refractive index WS_2_ nanostripes facilitates the excitation of photonic anomalies at the onset of the evanescence condition. As a consequence, light flow is effectively steered and trapped within the 2D‐TMDs heterostructures and the supporting dielectric slab. Photon harvesting is engineered in the flat optics regime by optimizing the thickness of the WS_2_ nanostripes, which serve as optical sensitizers. This innovative design achieves a resonant enhancement of optical absorption, up to a remarkable factor of 450%, when compared to a reference flat MoS_2_/WS_2_ heterostructure of equivalent thickness. This result highlights the promising potential of the novel 2D‐TMD platforms for scalable real‐world applications of van der Waals heterostructures, targeting photoconversion, photocatalysis, and energy storage.

## Graphical abstract

Large area fabrication of few‐layer MoS_2_‐WS_2_ vertical heterostructures nanoarrays over periodically nanostructured silica templates is demonstrated by means of a custom‐made physical deposition set‐up. The optical anomalies arising from subwavelength periodicity and the engraved WS_2_ nanostripes acting as optical sensitizers result in broadband and omnidirectional absorption enhancement.

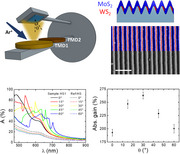

## 1 Introduction

2D materials and atomic van der Waals heterostructures have recently collected a strong scientific and technological interest due to their extraordinary properties, offering new solutions for device miniaturization down to the atomic scale.^[^ ^1^, ^2^, ^3^, ^4^, ^5^, ^6^, ^7^ ^]^ The family of 2D Transition Metal Dichalcogenides semiconductors (2D‐TMDs) has gained broad attention due to their tunable bandgap which is optimally matched to the solar spectrum, a promising feature in view of optoelectronic and energy conversion applications.^[^ ^8^, ^9^, ^10^, ^11^, ^12^, ^13^, ^14^, ^15^ ^]^

Owing to their 2D nature with atomically smooth surfaces and fully saturated chemical bonds, an additional opportunity to further engineer the optoelectronic response of a 2D device is offered by the combination of two different TMDs layers to form a van der Waals heterojunction, in which new optoelectronic properties arise from the combined band structure of the junction.^[^ ^16^, ^17^, ^18^, ^19^, ^20^, ^21^, ^22^ ^]^ Considering for instance the case of MoS_2_ and WS_2_, the coupling of their band structures gives rise to a type‐II heterojunction both in monolayer as well as in few‐layer regime with the valence band maximum belonging to WS_2_ and conduction band minimum belonging to MoS_2_.^[^ ^23^, ^24^ ^]^ As a consequence, photogenerated electron‐hole pairs are physically separated in opposite layers, thus increasing the carriers lifetime. In particular, TMD‐based van der Waals heterostructures are considered promising building blocks for the fabrication of self‐powered photodetectors and photocatalysts by exploiting the charge separation of the photogenerated carriers at the 2D interface without any external bias.^[^ ^25^, ^26^, ^27^, ^28^, ^29^, ^30^ ^]^

State‐of‐the‐art 2D‐TMDs photonic and optoelectronic devices – either based on a single material or a stack of multiple layers – typically rely on mechanically exfoliated single crystal flakes that are endowed with optimal optoelectronic properties.^[^ ^31^ ^]^ However, the exfoliation process poses several crucial limitations, mainly due to the small area of the 2D flakes limited at the micrometer scale, and to their random distribution on the sample surface with very poor control on the 2D material position and thickness.^[^ ^17^ ^]^ These limitations become even more stringent for the vertical or lateral assembly of van der Waals heterostructures, typically relying on controlled micro‐manipulation of the 2D flakes under an optical microscope.^[^ ^32^ ^]^ Due to these material issues, the nanofabrication of 2D‐TMDs heterostructure devices requires cumbersome and time‐consuming alignment procedures to localize and interconnect the micrometric flakes, recurring to low‐throughput nanolithography techniques.

The growth of 2D‐TMD layers over large area substrates is mandatory for the development of real‐world applications, and some fundamental challenges must be addressed for developing photonic devices at wafer‐scale based on such ultra‐thin materials. Large area growth techniques such as Chemical Vapor Deposition and Transport (CVD and CVT) have been recently developed, successfully demonstrating the growth of triangularly shaped 2D flakes grown over large areas via random reaction and precipitation of the vapor phase precursors at high temperatures.^[^ ^33^, ^34^, ^35^, ^36^ ^]^ The randomness of the volatile precursor and the high temperatures involved however prevent lithographic deposition of the TMD layers to form devices with controlled lateral arrangement.

Although the high absorption coefficient of few‐layer TMDs selects them as promising materials for photodetection and photoconversion applications, the overall photon absorption in the 2D regime is still low (≈10%) due to the extremely reduced optical path. Innovative ultrathin light trapping solutions effective at the nanoscale have been proposed for photo‐sensing and photoconversion applications.^[^ ^37^, ^38^, ^39^ ^]^ Plasmonic approaches have been explored by coupling noble metal nanoparticles to the active 2D material, showing enhancement of the photocurrent in a MoS_2_‐based field effect transistor via plasmonic near‐field.^[^ ^40^, ^41^, ^42^ ^]^ Another interesting phenomenon that can be exploited when plasmonic arrays are coupled to 2D‐TMDs is the hot‐electron injection into the semiconductor, though the efficiency gain is actually very poor.^[^ ^43^ ^]^

In order to avoid undesired ohmic losses typical of plasmonic nanostructures, alternative approaches exploiting all‐semiconductor nanophotonic schemes have been recently explored. Indeed, the high refractive index of the TMDs layers qualifies them as ideal candidates for light wavefront manipulation and enhanced light‐matter interaction in the flat optics regime.^[^ ^44^, ^45^, ^46^, ^47^ ^]^

In this work, we demonstrate a scalable nanofabrication approach for the maskless growth of large area (cm^2^ scale) TMDs confined onto transparent nanogrooved templates to form van der Waals heterostructures. An original physical deposition process based on the ion beam sputtering of bulk TMDs targets promotes the controlled growth of ultrathin homogeneous TMDs films and enables their engineering in a two‐step maskless process. By exploiting the nanogrooved templates, arrays of tilted nanostripes based on few‐layer WS_2_ are first grown by glancing angle deposition, and subsequently conformally coated by a continuous few‐layer MoS_2_ film, thus achieving large area van der Waals heterostructure nanoarrays.

On the one hand, the periodic van der Waals heterostructure nanoarrays promote the excitation of photonic anomalies under evanescent conditions, which steer light flow parallel to the ultrathin active material. On the other hand, the periodic modulation of the refractive index provided by the tilted WS_2_ nanostripes, which act as optical sensitizers, dramatically enhances photon absorption in the TMDs medium.

The enhancement of solar light harvesting in ultrathin van der Waals heterostructures, functionalized according to flat optics concepts, represents a promising step toward real‐world application of large area 2D‐TMDs heterostructures in photoconversion, nanophotonics, and energy storage applications.

## 2 Results and Discussion

### 2.1 Fabrication of Heterostructure Nanoarrays

**Figure 1:**
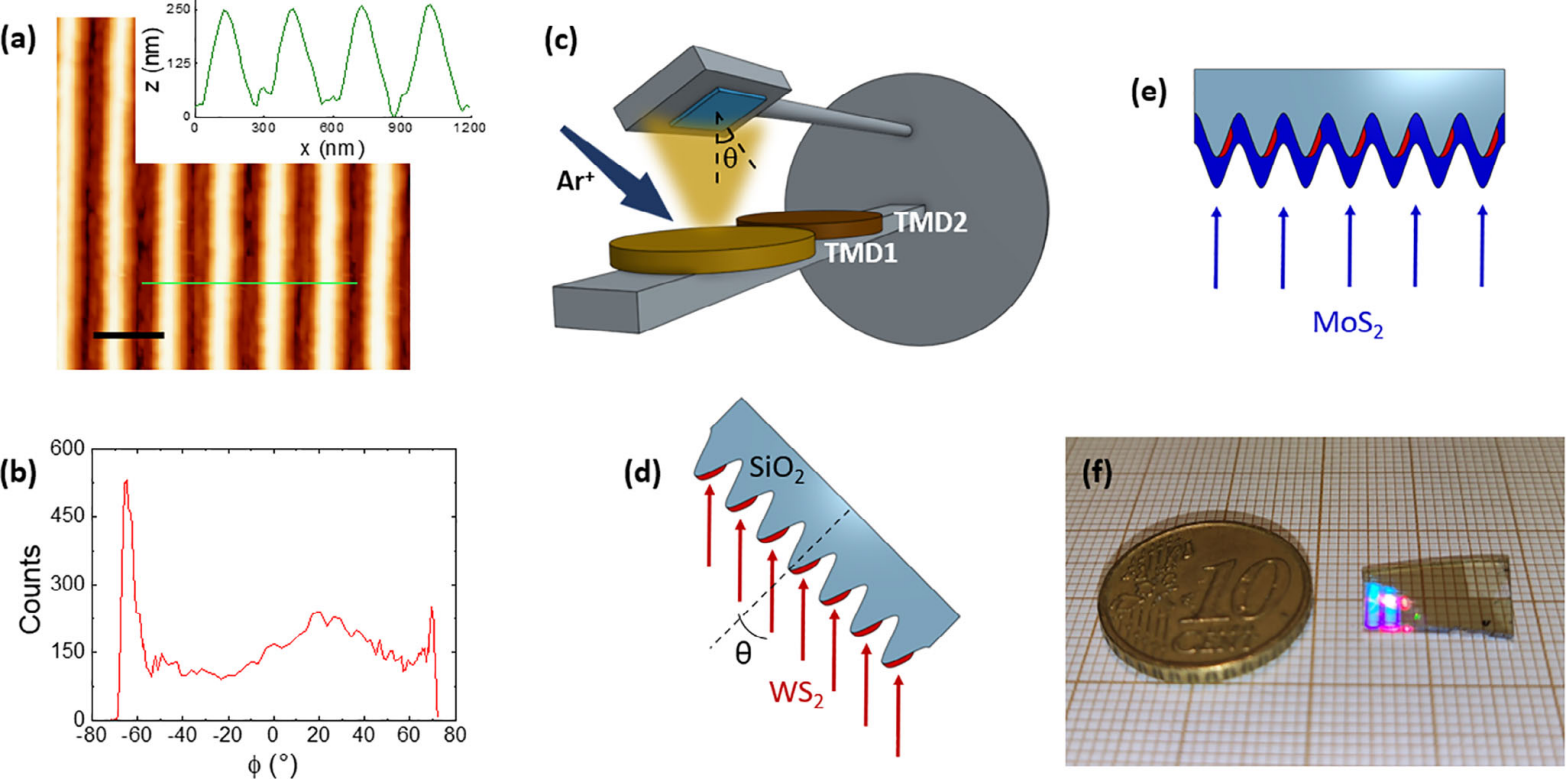
a) AFM image of a nanogrooved silica used as a template for the growth of large area heterostructures (the scale bar reads 400 nm); a line profile is extracted from the green line. b) Slope distribution of the template, as obtained from the derivative of the topography. c) Sketch for the IBS deposition set‐up with multiple TMD targets and rotating sample holder. d) Sketch for WS_2_ nanostripes maskless deposition at a glancing angle. e) Sketch for normal incidence deposition of a MoS_2_ capping layer. f) Picture of Sample 1, demonstrating large area nanopatterning and TMDs growth.

Periodic nanogrooved silica substrates have been fabricated recurring to a custom variant of the Laser Interference Lithography (LIL) coupled to Reactive Ion Etching (RIE).^[^ ^48^ ^]^ The characteristic morphology of the templates is investigated by means of Atomic Force Microscopy (AFM). **Figure** **1**a reports an AFM image together with an extracted cross‐section profile for one of the fabricated substrates, highlighting the high aspect ratio of our patterns. Specifically, the template considered in the following discussion is characterized by a vertical dynamic of about 250 nm and a periodicity of 300±15 nm, as assessed from the self‐correlation of the AFM image (see Figure S1, Supporting Information). The nanoridges morphology has been also studied in terms of the slope (*ϕ*) distribution, obtained from the first derivative of the AFM topography, and shown in Figure 1b. The two peaks in the slope distribution, observed at about *ϕ* = −65° and *ϕ* = 70°, arise from the steep facets defining the opposite sides of the nanoridges.

To fully exploit the potential of the high aspect‐ratio periodic templates for guiding the confined growth of self‐organized TMD nanostructures, we developed a custom Ion Beam Sputtering deposition set‐up that is schematized in Figure 1c. The sample holder manipulator allows to rotate the substrate at an angle *θ*, offering precise control on the formation of laterally disconnected TMD nanostructures confined on the illuminated facets by shadow deposition at glancing angles. Additionally, the set‐up has been designed to load multiple targets, thus enabling the sequential deposition and stacking of dissimilar TMD layers in a single experimental run. In this way, it is possible to obtain the fabrication of large area arrays of van der Waals heterostructures in a single maskless step.

As an example, Sample HS1 has been fabricated starting from the nanopatterned silica template characterized in Figure 1a,b. Deposition of WS_2_ at an off‐normal incidence angle *θ* = 50° on the high aspect‐ratio template leads to the formation of 9 nm thick laterally disconnected nanostripes, as a result of shadowing (red stripes in the cross‐section sketch of Figure 1d). In a second deposition step, we deposited a 3 nm thick MoS_2_ film at normal incidence onto the substrate, vertically stacking the conformal layer on top of the WS_2_ nanostripes. Remarkably, this approach further enables a variety of designs, from continuous conformal vertical heterostructures, to lateral heterojunction arrays by deposition of different TMDs on the two opposite facets, and finally to vertically stacked TMDs‐stripes.

After physical deposition at room temperature, the TMD layers adopt an amorphous phase (as revealed by the unstructured spectra observed both in optical extinction and in Raman spectroscopy, reported in Figure S2, Supporting Information). This is crucial for our stacking deposition approach: even if the IBS deposition is an energetic process, the deposition of the second layer does not damage the crystallinity of the first one because it is still in an amorphous condition. Only after deposition of the stacked bilayer, we proceed to recrystallize the TMD layers via high‐temperature annealing (750 °C) in a sulfur‐enriched atmosphere in order to preserve TMD stoichiometry, avoiding sulfur losses at high temperatures.^[^ ^49^ ^]^

The picture shown in Figure 1f represents Sample HS1 and the blue reflex in the illuminated left region of the sample is due to coherent scattering and diffraction from the highly periodic array of van der Waals WS_2_/MoS_2_ heterostructures. The result visually demonstrates the high throughput of our large‐area approach which leads to the fabrication of HS arrays over cm^2^ areas.

### 2.2 Sample Characterization

**Figure 2:**
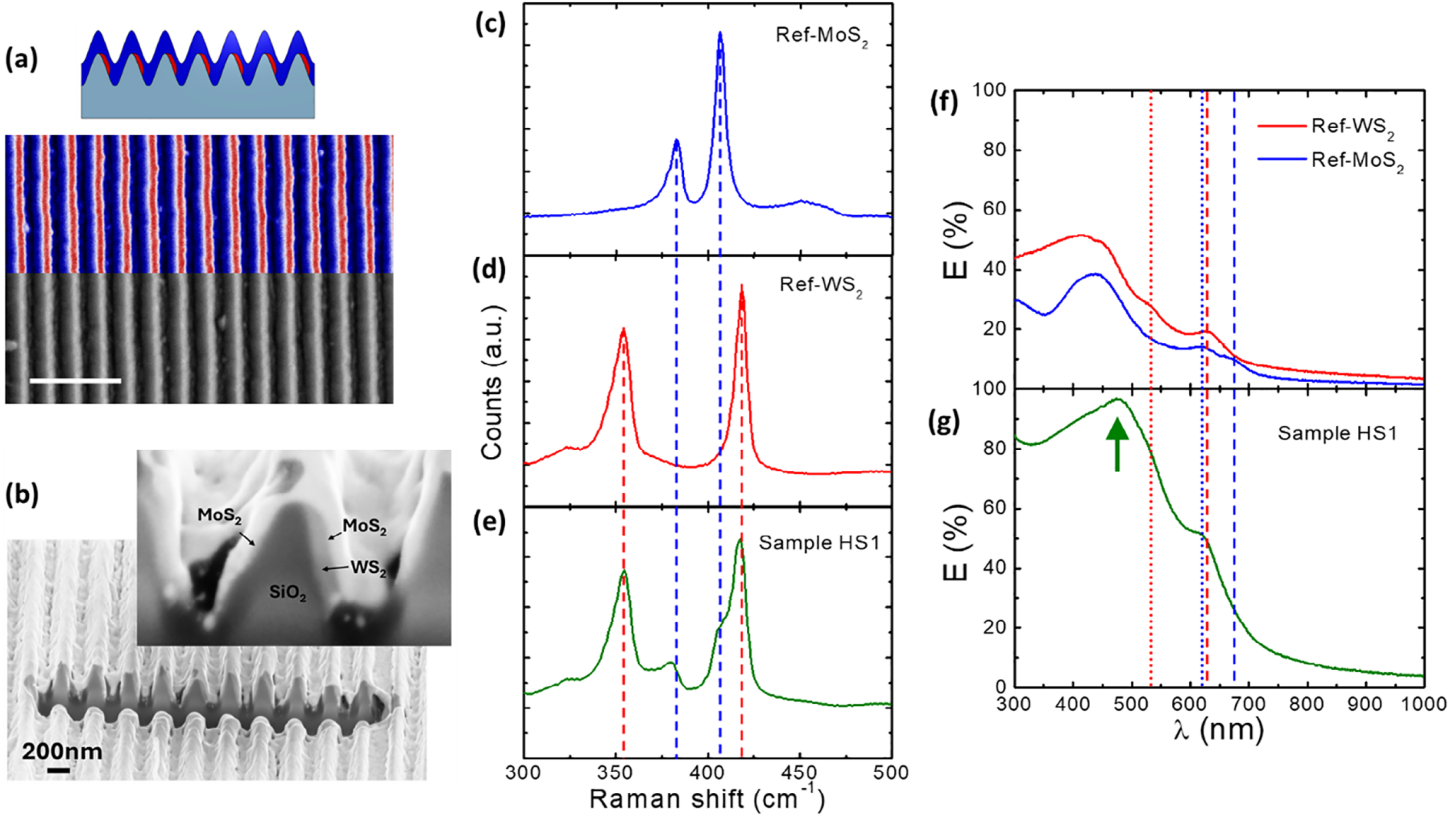
a) SEM image (backscattering BSE signal) of the heterostructures array Sample HS1 in false colors according to the sketch (scale bar reads 1 µm). b) Cross‐sectional HR‐SEM image of Sample HS1 along the FIB cut; inset shows a zoomed‐in image of a ripple, where the different materials are highlighted by the arrows (more details in Figure S3, Supporting Information). c–e) Raman spectra of reference samples MoS_2_ and WS_2_ compared to Sample HS1. f) Normal incidence optical extinction spectra for reference samples MoS_2_ and WS_2_ and g) of the heterostructure array HS (TE polarization).

Sample HS1 was characterized by means of SEM imaging, Raman micro‐spectroscopy, and optical spectroscopy. The SEM image in **Figure** **2**a – acquired using the Back Scattering Electron (BSE) signal – shows compositional contrast between the engraved WS_2_ nanostripes on the right side of the substrate nanoridges and the MoS_2_ signal on the left side, demonstrating that the maskless physical deposition process at glancing angles allows to obtain confinement of WS_2_ nanostripe arrays. The upper half of the SEM image is shown in false colors to match the cross‐section profile of the heterostructure (red for regions with higher BSE signal over the WS_2_ nanostripes, blue for regions with lower BSE signal over the MoS_2_ film). For a better understanding of the sample's inner structure, a cross‐sectional view was obtained by focused ion beam (FIB) milling and HR‐SEM imaging (see Methods). To reduce milling artifacts and prevent charging, a thin gold layer was deposited on top of the sample before proceeding with the FIB cut. The cross‐sectional image is reported in Fig. 2b and in Figure S3 (Supporting Information). A detailed view of a single ripple is shown in the inset, where the cross‐sectional structure can be indirectly inferred by the compositional contrast provided by the different secondary electron yields of MoS_2_, WS_2_, and SiO_2_ layers. As discussed in more detail further below, identification of the different material phases is instead provided by micro‐Raman spectroscopy.

In parallel with Sample HS1 fabrication, two flat reference samples were also fabricated to characterize the individual layers of WS_2_ and MoS_2_, namely Sample Ref‐WS_2_ and Sample Ref‐MoS_2_. The micro‐Raman spectra acquired for the reference flat TMD layers and the spectrum corresponding to the heterostructure nanoarray are shown in Figure 2c–e, respectively. The spectra acquired from Sample Ref‐MoS_2_ and Sample Ref‐WS_2_ (Figure 2c,d) are characterized by the Raman vibrational modes of MoS_2_ and WS_2_, thus confirming the growth of high‐quality TMD layers. The in‐plane mode E^1^_2_ _g_ and the out‐of‐plane mode A_1_ _g_ are located at 383 and 407 cm^−1^ for MoS_2_ and at 354 and 418 cm^−1^ for WS_2_, in good agreement with literature for the few‐layer regime close to the bulk value.^[^ ^50^, ^51^ ^]^ Colored dashed lines (blue for MoS_2_, red for WS_2_) are traced in correspondence to the E^1^_2_ and A_1_ _g_ modes of the reference samples, so to identify the Raman spectral signature of MoS_2_ and WS_2_ layers also in the spectrum acquired from Sample HS1 (Figure 2e). Flat MoS_2_ and WS_2_ films have been used as reference since possible strain effects induced by the templates on the TMD layers can be neglected, as demonstrated by the Raman spectra reported in Figure S4 (Supporting Information), showing negligible spectral shift due to strain for corrugated MoS_2_ films grown on similar silica gratings with respect to a flat layer. The combined presence of vibrational Raman modes belonging both to MoS_2_ and WS_2_ in Figure 2e independently confirms the formation of vertically stacked TMD heterostructures, as already suggested by the compositional contrast in HR‐SEM cross‐sectional images.^[^ ^52^, ^53^, ^54^, ^55^ ^]^

These references allow to conclude that our vertically stacked multilayer films are in a non‐intermixed phase, since the fingerprint of ternary alloy Mo_1‐x_W_x_S_2_ formation is not observed. In our case, the A_1_ _g_ peaks remain stable within experimental uncertainty (0.7 cm^−1^) around the values of pure MoS_2_ and WS_2_ phase respectively, while a shift of the A_1_ _g_ Raman mode by about15 cm^−1^ is observed when the composition shifts from pure MoS_2_ (x = 0) to pure WS_2_ (x = 1).

To further support this conclusion, in Figure S5 (Supporting Information) we show a comparison between two different flat heterostructures samples, one obtained by a single‐step recrystallization (after the bilayer deposition) and the other obtained by a double‐step recrystallization (the first after WS_2_ deposition, the second after MoS_2_ deposition). In both cases, the Raman spectra reported in Figure S5 (Supporting Information) exhibit negligible peak shifts (less than 0.7 cm^−1^) that are compatible with the experimental resolution, and well lower than the 15 cm^−1^ shift expected in the case of intermixing.^[^ ^52^, ^53^, ^54^, ^55^ ^]^

We also stress that the robustness and general validity of this conclusion, based on Raman spectroscopy, is not limited to the Mo‐W‐S ternary alloys but is also generalized to other dichalcogenide ternary alloys such as Mo_1‐x_W_x_Se_2_ (0 ≤ x ≤ 1).^[^ ^56^ ^]^

Comparable conclusions concerning the absence of alloying and intermixing after recrystallization have been reported in a similar experiment, where a vertical stack of metal precursors (W/Mo) of similar thickness in the nm range has been thermally annealed in sulfur‐rich atmosphere at temperatures in the range of 750 °C.^[^ ^57^ ^]^ In our case, since we deposit stoichiometric MoS_2_ and WS_2_ amorphous films which are characterized by strong metal‐sulfur covalent bonds, thermally induced mobility and interdiffusion in the 3D matrix are further reduced in comparison to the case of ref. [57]

A similar comparison between Sample HS1 with the reference TMD layers can be also done for the optical extinction in the VIS‐NIR spectral region. Figure 2f shows the optical extinction spectra at normal incidence for Sample Ref‐MoS_2_ and Sample Ref‐WS_2_ (blue and red curves), highlighting the excitonic modes of the two materials. A and B excitons are detected at λ = 673 and λ = 623 nm in the case of MoS_2_ and at λ = 631 and λ = 535 nm in the case of WS_2_, whereas band nesting spectral region is detected atλ ≈ 450 nm for both the materials, confirming the growth of TMD layers in the semiconducting 2H phase.^[^ ^58^, ^59^ ^]^ In ref. [58], the optical response of planar polycrystalline MoS_2_ films similar to the ones here discussed has been described effectively by an anisotropic dielectric tensor derived from the crystalline MoS_2_ film, since the layered polycrystalline domains are oriented with their c‐axis orthogonal to the substrate.

The spectrum of sample HS1, reported in Figure 2g, on one side shows the presence of the excitonic features of MoS_2_ and WS_2_ layers (the latter more pronounced due to the higher thickness) and on the other side shows a substantial increase of optical extinction in the whole spectral range, particularly relevant in correspondence to the excitonic features of the individual TMD components. Remarkably, we also highlight the appearance of a new prominent spectral feature – marked by the arrow – whose extinction increases up to 95% at ≈λ 480 nm. As discussed in the next section, such photonic anomaly dominates the optical response of the nanostructured HS array and is absent in the spectra of the planar MoS_2_ and WS_2_ reference samples (Figure 2f) which are characterized by the much weaker excitonic features. In turn, the photonic anomaly is not related to fine details of the polycrystalline grain distribution, but rather to the periodic spatial modulation of the TMD profile.

### 2.3 Photonic Anomalies

**Figure 3:**
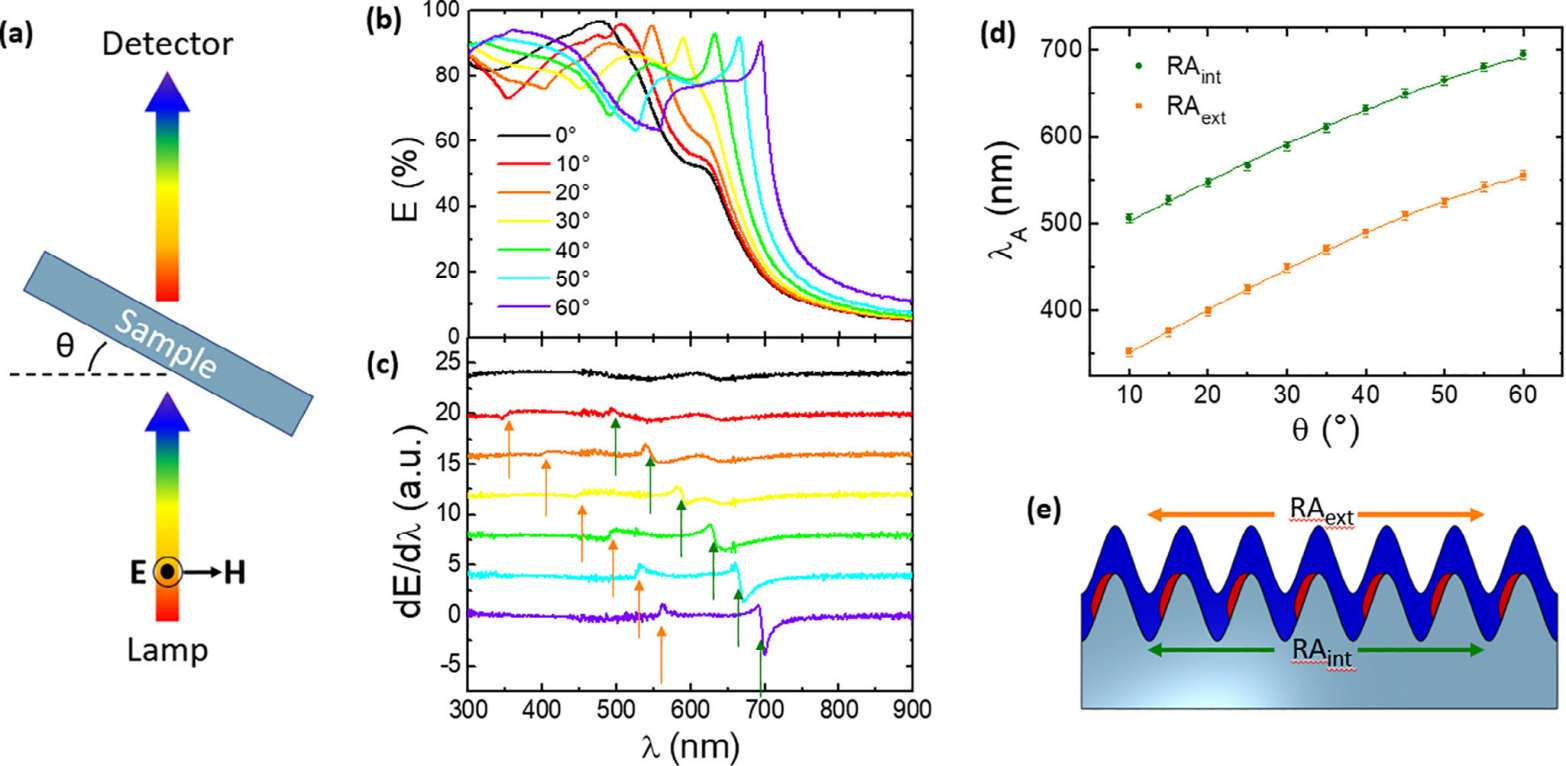
a) Schematics of angle‐resolved extinction measurements. b) Angle‐resolved extinction spectra for Sample HS1, referenced to a flat silica substrate. c) Derivatives of the spectra shown in panel (b). d) Dispersion of the Rayleigh anomalies as traced from the derivatives in panel (c). e) Sketch of the Rayleigh anomalies at the two different interfaces.

In order to attribute the nature of the photonic anomaly observed in the normal incidence extinction spectrum of Sample HS1, we performed a set of angle‐resolved extinction measurements recurring to a set‐up configuration as sketched in **Figure** **3**a. The spectra were acquired in S‐TE polarization, i.e., using linearly polarized light with the electric field oscillating perpendicular to the incidence plane and parallel to the nanostripes, by tilting the sample from *θ* = 0° to *θ* = 60° at steps of 5° according to the sketch shown in Figure 3a. For clarity, in Figure 3b we plot the angle‐resolved extinction spectra at 10° increments (the complete collection of spectra and its derivatives are plotted in Figure S6, Supporting Information). The intense extinction peak observed at ≈λ 480 nm for normal incidence illumination redshifts up to 700 nm as the incidence angle increases from 0° to 60°. Remarkably, we observe that the extinction in correspondence to this maximum is above 90% for every incidence angle. At shorter wavelengths an additional dispersive feature, corresponding to an asymmetric dip, redshifts from 350 to 550 nm as the tilt angle increases. Such pronounced extinction from the ultrathin 2D‐TMD nanostructured arrays is due to extraordinary light coupling in the flat optic regime and to the remarkably high dielectric constants of WS_2_ and MoS_2_ (*n* ≈ 5.5 and *k* ≈ 3 at wavelengths ≈500 nm).^[^ ^60^ ^]^

To better track the dispersion of these two spectral features, we calculated the first derivatives of the spectra, which are plotted in Figure 3c with an offset for better comprehension. The experimental dispersion data of the two photonic anomalies, identified by the wavelength for which the derivative is nulled, are shown in Figure 3d. The presence of two dispersive modes, originating from the periodic arrangement of 2D‐TMD nanoridges, can be tentatively attributed to the resonant excitation of Rayleigh Anomalies at the onset of the evanescent condition at the outer or inner interfaces of the 2D layers, and to the broadband excitation of guided mode anomalies confined at the inner interface of the grating. The dispersion of the Rayleigh anomalies can be described according to the following equation:^[^ ^61^ ^]^

(1) $$m\lambda =P\cdot \left(n_{eff}+n_{0}\sin\theta \right)$$

where *m* is the diffraction order, *P* is the grating periodicity, *n_eff_* is the effective refractive index at the evanescent condition between the external medium and nanostructured interface, *n*_0_ = 1 is the refractive index of the external medium (air in all the cases) and *θ* is the light incidence angle.

In Figure 3d we show a fit of the experimental dispersion data corresponding to the two photonic anomalies described in Equation 1, by setting *m* = 1 and *n*_0_ = 1 and by optimizing the free parameters *P* and *n_eff_*. The ± 5 nm experimental error bars on the wavelength data take into account the combined contributions arising from the detector spectral resolution and from the small angular errors of the goniometer. For the dispersive mode at shorter wavelengths (orange symbols), the free parameters of the fit lead to an effective refractive index *n_eff_* = 1.02 ± 0.04 and a periodicity *P_fit_* = 295 ± 7 nm. The result is in excellent agreement with the attribution to an external Rayleigh Anomaly (RA_ext_ sketch in Figure 3e), since the parameters are compatible with *n_air_* = 1 and with sample periodicity *P_AFM_* = 300 ± 15 nm assessed by the AFM characterization. For the dispersive mode at longer wavelengths (green symbols), the best fit of the effective refractive index is *n_eff_* = 1.66 ± 0.06 and of the periodicity is *P_fit_* = 274 ± 7 nm (still compatible at 1 σ with the AFM value). In this case the mode is Rayleigh anomaly propagating at the inner interface (RA_int_, sketch in Figure 3e) and is affected by the higher refractive index of the effective medium composed by the silica and the MoS_2_/WS_2_ heterostructures.

To study the influence of WS_2_ nanostripes as optical sensitizers, we fabricated additional heterostructure samples by varying the WS_2_ nanostripes thickness at fixed MoS_2_ thickness on nanostructured silica templates with morphology equivalent to Sample HS1. Sample HS2 was thus fabricated with halved WS_2_ thickness (≈4.5 nm) and Sample HS0 with no WS_2_ at all.

**Figure 4:**
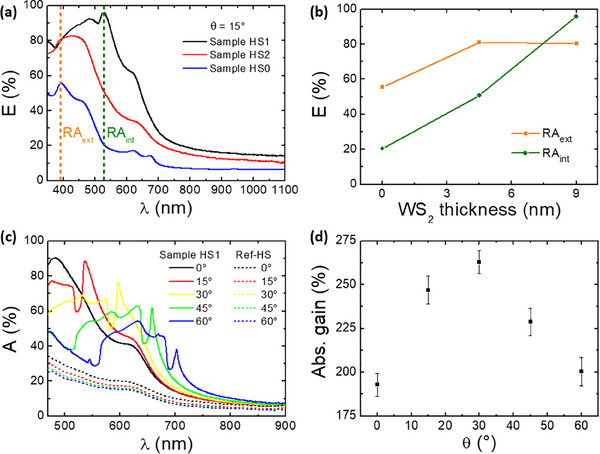
a) Extinction spectra at a fixed angle (*θ* = 15°) for Sample HS1 (black curve), Sample HS2 (red curve), and Sample HS0 (blue curve), referenced to the signal in the air. b) Extinction variation as a function of the WS_2_ thickness, in correspondence to the Rayleigh anomalies at *θ* = 15° respectively at λ = 530 nm for RA_int_ and λ = 390 nm for RA_ext_. c) Absorption spectra at different angles for Sample HS1 compared to Ref‐HS. d) Absorption gains in the spectral range 470–750 nm as a function of the incidence angle for Sample HS1 compared to Ref‐HS.

In **Figure** **4**a we report the optical extinction spectra acquired at a fixed angle *θ* = 15° for HS0 (blue curve), HS1 (black curve), and HS2 (red curve). The angle *θ* = 15° was chosen in order to better evidence the Rayleigh anomalies, avoiding spectral overlap with the TMDs excitonic features. As expected, we notice that the optical extinction of the heterostructures increases with the total thickness of the WS_2_ nanostripes. Additionally, we highlight that the extinction spectrum of sample HS0 shows the typical excitonic features of MoS_2_ along with a dispersive feature at ≈ = λ 390 nm that corresponds to the external Rayleigh anomaly, marked by the orange dashed line. The internal Rayleigh anomaly at ≈ = λ 530 nm is not visible in the extinction spectrum as it can be only identified in the derivative spectrum reported in Figure S8a (Supporting Information). If thin WS_2_ nanostripes are added to form heterostructures (Sample HS2), the optical response becomes immediately dominated by the WS_2_ excitonic features, and a very weak photonic anomaly can be distinguished both in the extinction spectrum and in the derivative spectrum reported in Figure S8b (Supporting Information). When the WS_2_ thickness is further increased (Sample HS1), the internal Rayleigh anomaly, marked by the green dashed line, dominates the optical extinction.

In Figure 4b we summarize the observed trend of the optical extinction as a function of the WS_2_ thickness in the TMD heterostructures in correspondence to the inner/outer Rayleigh anomalies for *θ* = 15°. Remarkably, the extinction in correspondence to the inner anomaly, which is not observable in the bare MoS_2_ film, reaches a value as high as 95% in the presence of the thick WS_2_ nanostripes which thus function as optical sensitizers, enhancing light confinement inside the active TMD layer. On the other hand, the contribution to extinction from the outer anomaly, which steers light at the TMD/air interface, is already high in the case of simple MoS_2_ corrugated films (extinction for HS0 sample is in the range of 60%)^[^ ^48^ ^]^ and increases just slightly up to 80% when the WS_2_ nanostripes are combined to form heterostructures.

A complementary investigation of the Raman dependence from the thickness of WS_2_ and MoS_2_ layers in different MoS_2_/WS_2_ heterostacks is discussed in Figure S7 (Supporting Information), demonstrating the reproducibility and control on the relative TMD thickness of our growth process.

### 2.4 Optical Absorption Enhancement Characterization

Both scattering and absorption contribute to the optical extinction features detected in Figure 3. In order to precisely identify the contribution to the optical absorption of the outer/inner optical anomalies, and to unambiguously determine the light‐harvesting performance of the nanostructured van der Waals heterostructures, we performed integrated absorbance measurements. For this purpose, we developed a custom‐modified integrating sphere set‐up, shown in Figure S9 (Supporting Information) and described in the relative Methods section, which allows to record absorption spectra of the samples placed inside the sphere.

In Figure 4c we compare the optical absorption of the periodic array of heterostructures for Sample HS1 at different illumination angles (solid lines) with that of a reference flat bilayer stack of WS_2_ and MoS_2_ of equivalent thicknesses, namely Sample Ref‐HS (dashed lines). The absorption spectra of Sample HS1 show both the characteristic narrowband optical anomalies dispersing across the investigated spectral range for increasing incidence angle and a broadband enhancement of absorption due to incoherent light scattering and waveguiding in the dielectric slab.^[^ ^47^ ^]^ In the case of the reference flat HS both narrowband and broadband absorption enhancement are absent despite the amount of optically absorbing TMD material is the same, since energy‐momentum conservation rules prevent photon coupling and steering into the flat dielectric slab. In particular, we stress the extraordinary absorption enhancement for Sample HS1 with respect to the flat reference sample, which exceeds 450% at 15° incidence angle at the resonant wavelength ≈530 nm corresponding to excitation of the inner Rayleigh anomaly (see Experimental Section for further details). In **Table** **1** we compare this result with previous works on nanostructured MoS2 layers, clearly highlighting the remarkable record enhancement achieved with our HS arrays fabricated at a glancing angle.

**Table 1:** Comparison of the maximum optical absorption enhancement for different configurations of nanostructured TMD layers (for each case, the enhancement is referred to as a flat reference sample covered with the same active layers).

Active layer configuration	Max optical absorption enhancement	Refs.
TMDs HS nano‐arrays WS2 (9nm) – MoS2 (3nm) Conformal Amorphous MoS2 (30nm) on polymer grating Conformal MoS2 (3.2 nm) on silica grating MoS2 nanostripes (3.2 nm) on flat silica	450% 300% 240% 375%	This work ^[^ ^62^ ^]^ ^[^ ^48^ ^]^ ^[^ ^47^ ^]^

A further relevant issue to be highlighted in view of real‐world light harvesting applications based on ultrathin TMD materials is the omnidirectional enhancement of the optical absorption when the illumination angle *θ* is varied in the range 0°– 60°, being such conditions indeed typical of non‐concentrated solar applications in flat‐plate absorbers. In order to quantitatively assess the latter feature, in Figure 4d we define the absorption gain *G* (averaged in the spectral range 470–750 nm) as the difference between the absorption of the nanostructured sample *A_HS_* and the absorption of the flat reference stack *A_ref_*, normalized to the latter according to *G* = (*A_HS_* – *A_ref_*)/*A_ref_*. For near‐normal incidence angles the absorption gain of the heterostructure array reads about 180%, further increasing for larger angles with a maximum of about 260% for *θ* = 30°. Such results are remarkable in view of photoconversion applications based on ultrathin TMD materials, since critical angular alignment with respect to the light source is no longer required in the flat optic scheme here addressed. The maximum absorption gain observed for non‐normal incidence angles *θ* ≈30° is related to the tilted geometry of the WS_2_ nanostripes which are supported on the steep SiO_2_ facets (see Figure 1b,d).

By carefully matching the periodicity of the template with the extraordinary dielectric constant of the TMD layers, our approach allows to engineer the optical absorption across a broad spectral range of interest for solar energy harvesting. The flat optics functionality of the WS_2_ nanostripe array is manifested in two complementary ways: omnidirectional and broadband light harvesting performance arises from the light scattering and waveguiding into the dielectric slab, while resonant narrowband amplification of absorption is due to Rayleigh photonic anomalies which steer light at the onset of the evanescence condition. The accurate control on the TMD growth condition, combined with the engineering of the faceted template morphology, offers a straightforward route for the fabrication of tailored TMD heterostructure designs tilted out‐of‐plane, at variance with state‐of‐the‐art lithographic techniques which are limited to planar configurations. We stress out that in the case of layered materials such as TMDs, characterized by a strong optical anisotropy, a slope‐selective fabrication process of the nanostructured substrate enables out‐of‐plane tilt of the material at a specific angle, therefore offering the possibility to finely control the optical response of the metasurface. Such tilted TMD configurations are also of potential interest in nanophotonic devices aiming at polarization and directional control of light emission.^[^ ^58^, ^63^, ^64^ ^]^

The peculiarity of the periodic faceted templates ensures simple and maskless nanofabrication of 2D‐TMD heterostructure nanoarrays extending over cm^2^ areas. The high‐refractive index WS_2_ nanostripes function as an optical sensitizer media that boosts optical absorption in comparison both to the single MoS_2_ layer and to the equivalent flat reference WS_2_/MoS_2_ bilayer. The vertical stacking of type‐II heterostructures with their staggered alignment of energy levels represents a crucial step toward the optimization of 2D‐TMD‐based self‐powered photoconversion devices aiming, e.g., at solar energy harvesting, photocatalytic photodissociation of polluting dyes in solution or energy storage.^[^ ^62^, ^65^ ^]^ We stress out that upscaling of the process to wafer scale can be readily accomplished by adopting industrial scale interference lithography set‐ups and sputtering – deposition equipment.

## 3 Conclusion

We propose an original maskless deposition process for the fabrication of large‐area nanoarrays of TMD‐based van der Waals heterostructures. Nanostructured silica templates were patterned via laser interference lithography, forming uniaxial nanogrooves engineered with a high aspect‐ratio faceted profile. Laterally confined WS_2_ nanostripe arrays are obtained in a single maskless step over cm^2^ areas by selective shadow deposition of WS_2_ nanostripes at glancing angles, followed by conformal MoS_2_ deposition. By varying the thickness of WS_2_ facets, template periodicity, and photon incidence angle, the light‐matter properties of the TMDs heterostructures can be engineered systematically, steering light flow at the evanescence condition.

In particular, we demonstrate the role of the WS_2_ nanostripes as optical sensitizers in the flat optic regime, boosting the contribution of photonic Rayleigh anomalies confined at the inner TMD interface. In this way, an absorption gain exceeding 450% is obtained when the resonant condition of the Rayleigh anomaly is met, by optimizing the periodicity of the HS nanoarray and the illumination angle. Moreover, integrating over the whole visible spectrum we demonstrate a broadband and omnidirectional absorption gain as high as 260% with respect to a flat reference template of equivalent TMD composition, due to the excitation of guided modes supported by the dielectric template.

The successful integration of dissimilar 2D‐TMDs layers over faceted nanogrooved templates enables the straightforward formation of type‐II van der Waals heterostructures over large area templates, which represent the fundamental building blocks required for self‐powered photoconversion applications. These results pave the way for the realization of real‐world 2D devices promoting photon harvesting for a broad range of applications from energy conversion and storage to sensing and quantum technologies.

## 4 Experimental Section

### Templates Fabrication

A positive photoresist layer (AZ MIR 701 by MicroChemicals) was spin‐coated on polished fused silica substrates. Using a Lloyd's mirror configuration and a 405 nm laser diode, Laser Interference Lithography was employed to impress an interference pattern on the resist layer. The pattern was then transferred to the silica substrate by use of a Reactive Ion Etching process based on CF_4_ gas, finally resulting in nanogrooved silica substrates with high aspect ratio structures.

### Large Area 2D TMD Growth

TMD layers were grown by a physical deposition process based on Ion Beam Sputtering of bulk targets. The substrate was loaded in a UHV chamber filled with argon to the operative pressure of 6∙10^−4^ mbar. A Tectra RF plasma gun was used to irradiate the TMD target with an energetic ion beam (1,44 keV), resulting in the sputtering deposition of the material on the substrate. The process was calibrated by use of a quartz microbalance. A customized manipulator allows to load multiple TMD targets to perform sequential stacked deposition and to tilt the substrate to perform deposition at an off‐normal angle. By combining this with the use of nanostructured templates as substrates, the maskless deposition of large‐area nanoarrays of TMD‐based van der Waals heterostructures was achieved by exploiting shadowing effects at off‐normal deposition angles.

The deposited layers are then recrystallized at 750 °C in a sulfur‐enriched atmosphere. To this end, a tubular furnace with a 2‐inch quartz tube was employed. The sample was placed at the center of the furnace and a quartz boat containing sulfur powder was placed upstream in a lower temperature region of the furnace. A 100sccm argon flux was used as a carrier to transport sulfur to the sample.

### Atomic Force Microscopy (AFM) Imaging

The AFM images shown in this work were acquired using a NanoMagnetics ezAFM operating in tapping mode, equipped with high aspect ratio silicon tips supplied by NANOSENSORS. The images were processed using the freeware scanning probe microscopy software WSxM.^[^ ^66^ ^]^

### Scanning Electron Microscopy (SEM) imaging

Top‐view SEM images of the large area heterostructures array were acquired in back‐scattered electrons signal using a Hitachi SEM SU3500. The thermionic electron source was biased at 10 kV for proper imaging.

### Focused Ion Beam (FIB) milling and HR‐SEM imaging

Cross‐section milling and imaging were performed using the CrossBeam 1540xb by Zeiss. The imaging was done by setting the accelerating voltage at 4 keV and using an in‐lens detector. Before the FIB milling, the sample was covered by 50 nm of thermally deposited gold to protect the TMD layers during milling and minimize charging effects during imaging.

### Micro‐Raman characterization

The vibration properties of the fabricated samples are investigated by use of a NRS‐4100 Raman microscope (JASCO) operating in back‐scattering operation, or by using a Horiba Raman XploRA microscope in the same configuration. Measurements are performed with a green laser (532 nm) and a 100x objective lens. The laser power was kept low to avoid damaging the sample, and 2400 grooves per millimeter grating is used to achieve the best instrumental resolution.

### Optical Extinction Measurements

The optical properties are studied by means of angle‐resolved extinction spectroscopy. A compensated halogen‐deuterium lamp is used as the light source to illuminate the samples in the wavelength range 300–1100 nm and the signal was detected by an Ocean Optics HR4000 spectrometer. Light was coupled to optical fibers with a core diameter of 600µm. The sample was mounted on a rotating stage that allows to control of the tilt angle *θ* (from normal incidence 0° to 60°) and placed in the middle of the transmission line.

### Total Integrated Absorption Measurements

A Thorlabs 4P4 Integrating Sphere was fiber coupled to a collimated supercontinuum white laser (SuperK COMPACT – NKT Photonics) through a polarizer and optical filters. A custom sample holder was machined via 3D printing to allow the rotation of the sample inside the sphere, therefore enabling angle‐resolved illumination. Integrated absorption measurements could thus be performed under the same excitation conditions employed for the angle‐resolved transmission measurements shown in Figure 3. The absorption gains were always calculated by subtracting a few % background offset, corresponding to the value at 900 nm wavelength where TMDs do not have optical transitions.

## Conflict of Interest

The authors declare no conflict of interest.

## Supporting information



Supporting Information

## Data Availability

The data that support the findings of this study are available from the corresponding author upon reasonable request.
