# Supplementary material for: Maskless Synthesis of van der Waals Heterostructure Arrays Engineered for Light Harvesting on Large Area Templates
Source: Small. 2025 Mar 5;21(15):2400943. doi: 10.1002/smll.202400943 (PMC12001309; doi:10.1002/smll.202400943)
Supplement: Supplementary file 1 — Supporting Information [file SMLL-21-2400943-s001.docx]

Supporting Information

Maskless synthesis of van der Waals heterostructure arrays engineered for light harvesting on large area templates

M. Gardella, G. Zambito, G. Ferrando, L. Ferrari Barusso, R. Chennuboina, L. Repetto, M. Barelli, M. C. Giordano, F. Buatier de Mongeot*

**Figure SI1: templates morphology analysis**


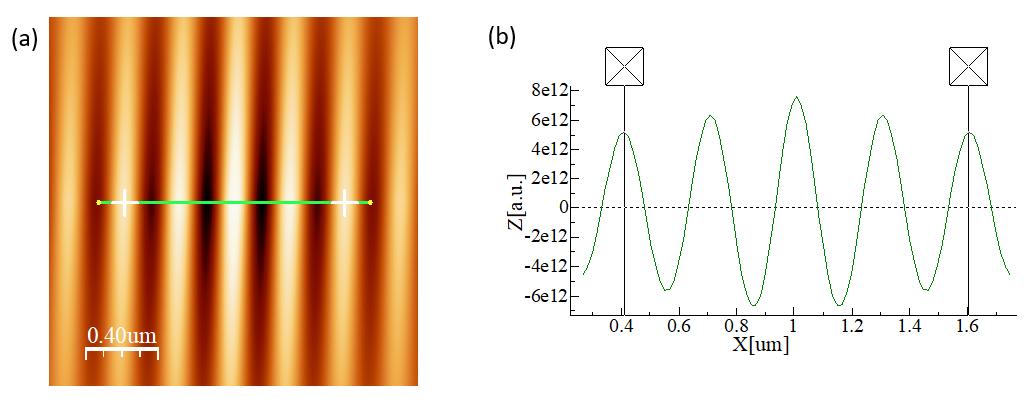


a) Self-correlation map of the topography image shown in Figure 1a; b) cross-section profile extracted from the green line of panel *a*, resulting in a periodicity of 300nm; an uncertainty of about 15 nm on the periodicity is taken into account as a consequence of the AFM tip resolution.

**Figure SI2: as deposited TMD layers**


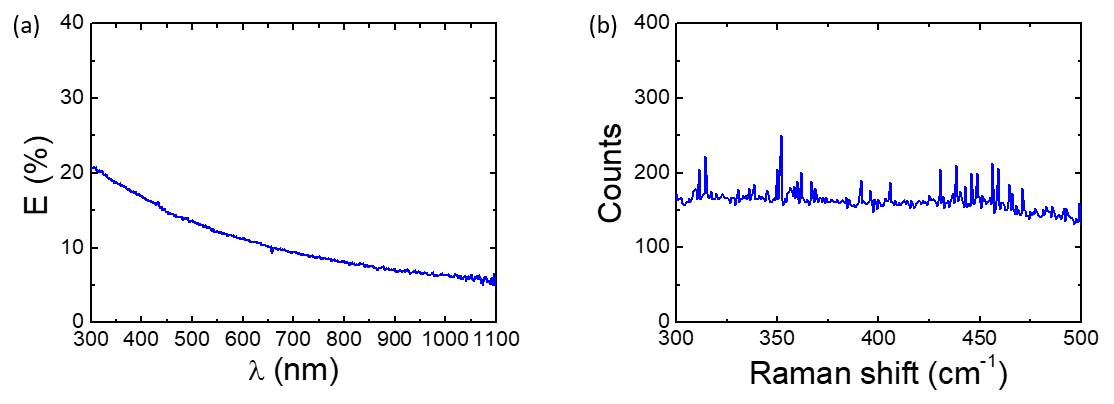


Optical and vibrational characterization for as-deposited MoS_2_ layers, showing unstructured response typical of the amorphous phase: no excitonic features can be found in the extinction spectrum (a), neither characteristic vibrational modes in the Raman spectrum (b).

**Figure SI3: cross-sectional HR-SEM image**

**
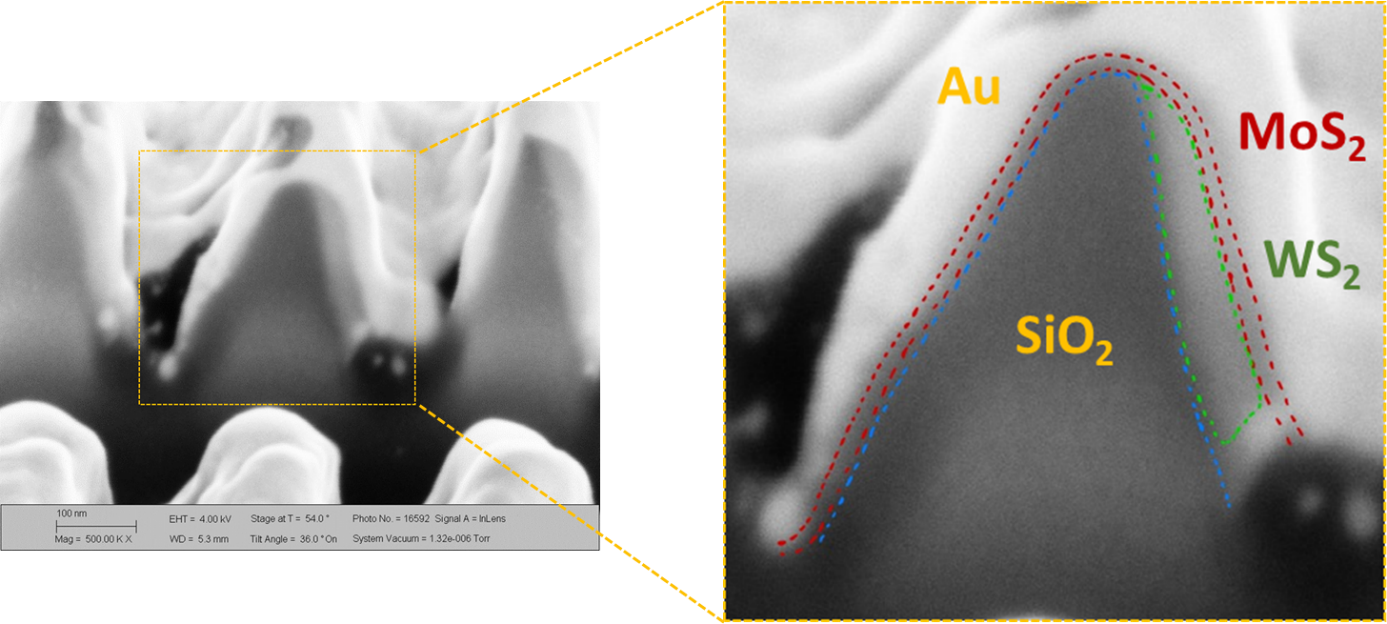
**

Zoomed-in image of the cross-sectional HR-SEM image with colored blue, red and green dashed lines for SiO_2_, MoS_2_ and WS_2_ respectively.

**Figure SI4: strain**

Raman spectra acquired for MoS_2_ layers grown on a flat substrate and on silica grating with increasing average curvature. No clear shift is detected in the MoS_2_ vibrational modes, therefore excluding strain related shifts.

**Figure SI5: intermixing**

Raman spectra acquired on two different heterostructures samples with equivalent thicknesses, one realized in a single step process (i.e. WS_2_ deposition – MoS_2_ deposition – recrystallization) and the other realized in a dual step process (i.e. WS_2_ deposition – MoS_2_ deposition – recrystallization). Being the E modes unshifted and being the A modes shift comparable with the experimental resolution, intermixing of the TMD layers can be excluded.

**Figure SI6: Sample HS1 angle resolved extinction measurements**


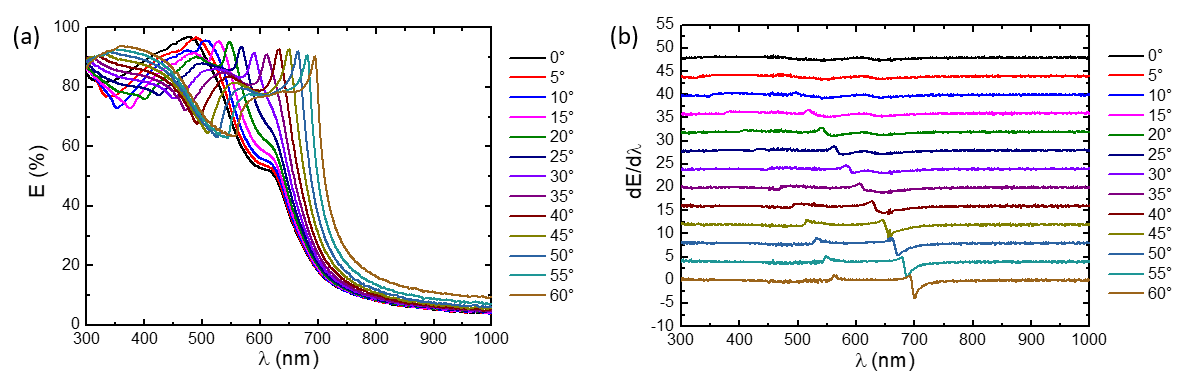


a) Angle resolved extinction spectra acquired for Sample HS1 at 5° steps; b) derivatives of the spectra shown in panel *a*, clearly showing the presence of two dispersive modes.

**Figure SI7: thickness dependent characterization**


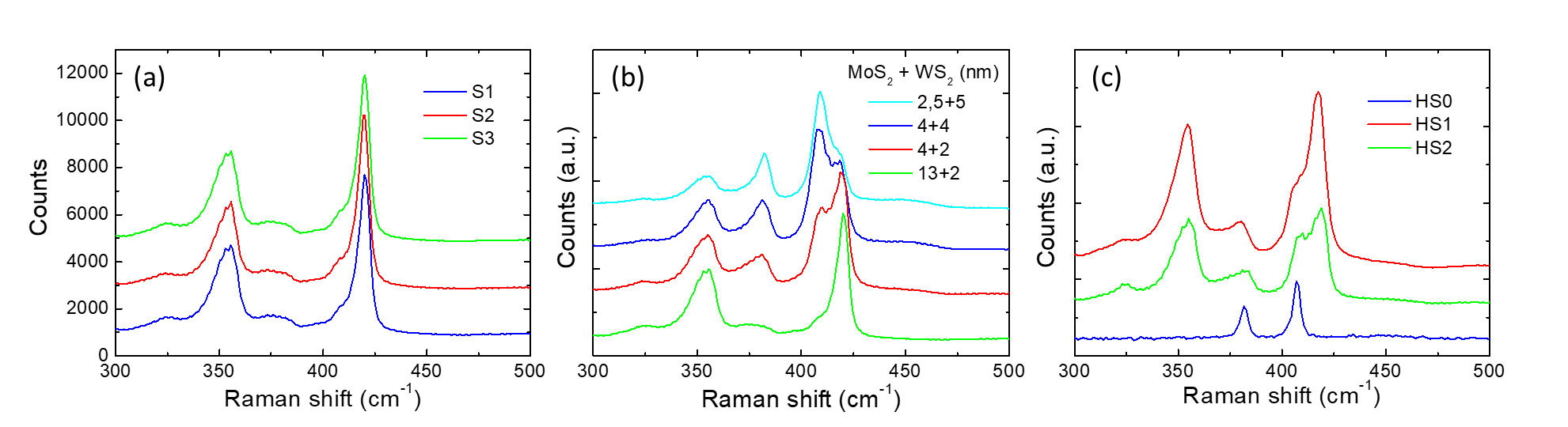


(a) Raman spectra of three different HS samples with the same nominal WS_2_/MS_2_ thickness 13/2 nm, demonstrating growth reproducibility; (b) Raman spectra of WS_2_/MoS_2_ flat heterostructures with different WS_2_/MoS_2_ thickness ratio, which is proportionally reflected by the signal ratio between the two materials; (c) Raman spectra comparison between samples HS0, HS1 and HS2, showing a similar proportionality between the signal and the WS_2_/MoS_2_ thickness ratio.

**Figure SI8: Sample HS0 and Sample HS2 derivative spectra**


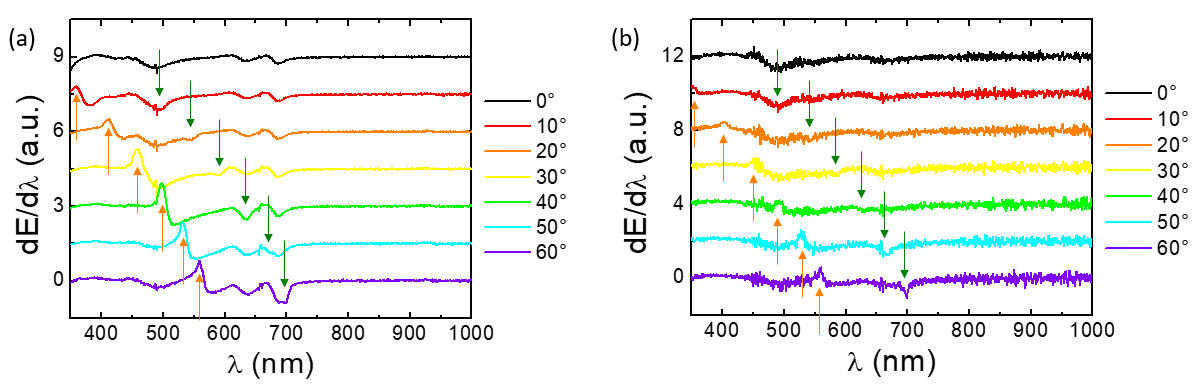


Derivatives of the angle resolved extinction spectra acquired for a) Sample HS0 and b) Sample HS2. Orange and green arrows are used to track the dispersion of the external Rayleigh anomaly and internal Rayleigh anomaly, respectively.

**Figure SI9: schematic of the integrating sphere**


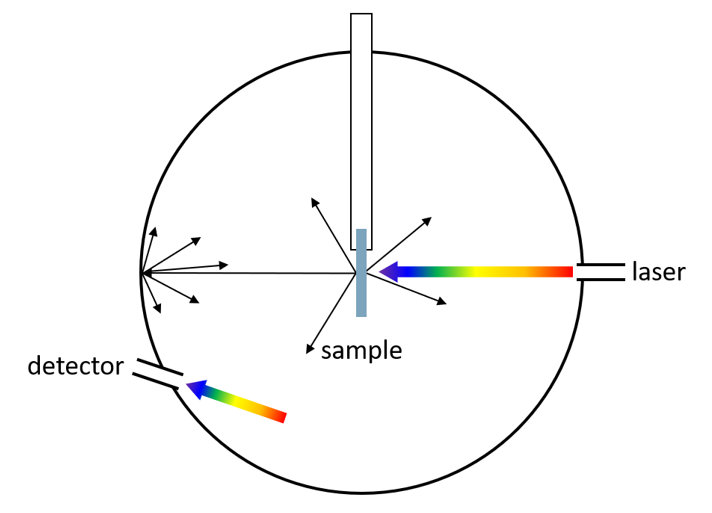


Schematics of the integrating sphere and customized sample holder, which can be rotated to perform angle resolved absorption measurements and shifted to remove the sample from direct beam.
